# DNA Barcodes of Asian Houbara Bustard (*Chlamydotis undulata macqueenii*)

**DOI:** 10.3390/ijms13022425

**Published:** 2012-02-22

**Authors:** Ibrahim A. Arif, Haseeb A. Khan, Joseph B. Williams, Mohammad Shobrak, Waad I. Arif

**Affiliations:** 1 Prince Sultan Research Chair for Environment and Wildlife, Department of Botany and Microbiology, College of Sciences, King Saud University, Riyadh 11451, Saudi Arabia; E-Mail: iaarif@hotmail.com; 2 Department of Evolution, Ecology and Organismal Biology, Ohio State University, Columbus, OH 43210, USA; E-Mail: williams.1020@osu.edu; 3 Department of Biology, College of Science, Taif University, Taif 5700, Saudi Arabia; E-Mail: mshobrak@gmail.com; 4 Ibn Khaldun International School, Riyadh 11321, Saudi Arabia; E-Mail: waad05aarif@hotmail.com

**Keywords:** DNA bar-coding, houbara bustard, cytochrome oxidase, gene sequencing, phylogenetics

## Abstract

Populations of Houbara Bustards have dramatically declined in recent years. Captive breeding and reintroduction programs have had limited success in reviving population numbers and thus new technological solutions involving molecular methods are essential for the long term survival of this species. In this study, we sequenced the 694 bp segment of COI gene of the four specimens of Asian Houbara Bustard (*Chlamydotis undulata macqueenii*). We also compared these sequences with earlier published barcodes of 11 individuals comprising different families of the orders Gruiformes, Ciconiiformes, Podicipediformes and Crocodylia (out group). The pair-wise sequence comparison showed a total of 254 variable sites across all the 15 sequences from different taxa. Three of the four specimens of Houbara Bustard had an identical sequence of COI gene and one individual showed a single nucleotide difference (G > A transition at position 83). Within the bustard family (Otididae), comparison among the three species (Asian Houbara Bustard, Great Bustard (*Otis tarda*) and the Little Bustard (*Tetrax tetrax*)), representing three different genera, showed 116 variable sites. For another family (Rallidae), the intra-family variable sites among the individuals of four different genera were found to be 146. The COI genetic distances among the 15 individuals varied from 0.000 to 0.431. Phylogenetic analysis using 619 bp nucleotide segment of COI clearly discriminated all the species representing different genera, families and orders. All the four specimens of Houbara Bustard formed a single clade and are clearly separated from other two individuals of the same family (*Otis tarda* and *Tetrax tetrax*). The nucleotide sequence of partial segment of COI gene effectively discriminated the closely related species. This is the first study reporting the barcodes of Houbara Bustard and would be helpful in future molecular studies, particularly for the conservation of this threatened bird in Saudi Arabia.

## 1. Introduction

**Figure 1:**
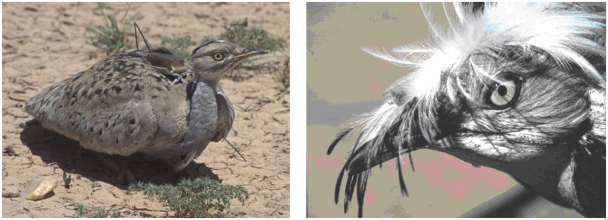
Asian Houbara Bustard (*Chlamydotis undulata macqueenii*).

Houbara Bustards (*Chlamydotis undulata*; Jacquin, 1784) are small to mid-sized birds, measuring 55–65 cm from beak to tail and with wingspans of 135–170 cm. Males weigh 1.15–2.4 kg and females 1–1.7 kg. Three subspecies of Houbara bustard have been recognized: *Chlamydotis undulata fuertaventurae*, resident in the Canary Islands; *Chlamydotis undulata undulata*, resident or locally dispersive in North Africa; and *Chlamydotis undulata macqueenii* (Figure 1), the Asiatic subspecies, resident in southern parts of its breeding range from the Arabian Peninsula across to the Indian subcontinent, but migratory in Central Asia [1]. Based on morphological evidence, some reports argue that the subspecies *C. u. macqueenii* should be considered as a full species, Macqueen’s Bustard (*Chlamydotis macqueenii*). However, more extensive genetic studies are required before a unanimously acceptable taxonomic classification can be achieved. Houbara Bustards breed in deserts and other arid sandy areas. North African and Arabian populations may be sedentary or partially migratory, moving relatively short distances to find food; populations from Turkmenistan east to China are migratory, and winter in large numbers in India, Pakistan, Iran and parts of the Middle East.

Houbara Bustards have undergone rapid population declines over the last three decades as a result of widespread hunting and loss of habitat; they have been classified as “Vulnerable” by the International Union for Conservation of Nature (IUCN). It has been traditionally hunted by falconers throughout Arabic countries, resulting in a marked reduction of houbara populations in these regions, especially in Saudi Arabia [2]. The National Commission for Wildlife Conservation and Development (NCWCD) (now renamed the Saudi Wildlife Authority) in Saudi Arabia indicated its intention to draft an international agreement and management plan with the aim of consolidating efforts to conserve the houbara throughout its range [3]. Several conservation measures, including the establishment of protected areas, education of the public, restriction of hunting, captive breeding and reintroduction have been undertaken by NCWCD [3]. A critical review of the houbara program emphasized the need for field studies, public-awareness programs and international collaboration, in addition to captive-rearing and release [3]. The first re-introduction of captive-bred Houbara Bustard took place in 1992 in the Mahazat as-Sayd protected area in central Saudi Arabia with the support of the National Wildlife Research Center (NWRC) [4]. By the end of 2011, the total numbers of adult Houbara Bustards exclusively dedicated to the captive breeding program at NWRC were 629 (217 males and 412 females). However, captive breeding programs have had limited success in restoring population numbers and thus radical technological solutions involving molecular methods are essential for the long-term survival of the species [5]. Recent advances in conservation genetics and the emergence of molecular markers have shown potential as an aid for the success of captive breeding programs [6–9].

DNA barcoding using mitochondrial cytochrome oxidase subunit I (COI) sequences has the potential for discriminating closely-related species across diverse phyla in the animal kingdom [10,11]. Kerr *et al*. [12] have demonstrated that DNA barcoding can be effectively applied across the geographical and taxonomic expanse of bird species. Even a single DNA barcode has been suggested as a rapid tool to discover monophyletic lineages within a metapopulation that might represent undiscovered cryptic species [13]. For evaluating the discriminatory power of a barcode, it would be more appropriate for all members of a genus be examined, rather than a random sample of imprecisely defined close relatives, and taxa to be included from more than one geographic region [14]. In recent years, DNA barcoding has been utilized for species identification of birds from different regions of the world [12,15–19]. However, the barcodes of Saudi Arabian birds have been reported for only a few species [20,21].

In the present investigation, we have sequenced the 694 bp region of COI gene of Houbara Bustard and compared these sequences with previously published sequences of different species. We have established a genetic barcode for Houbara Bustards that can be used in the future for various purposes, such as species identification, molecular diversity analysis and conservation planning.

## 2. Results

**Figure 2:**
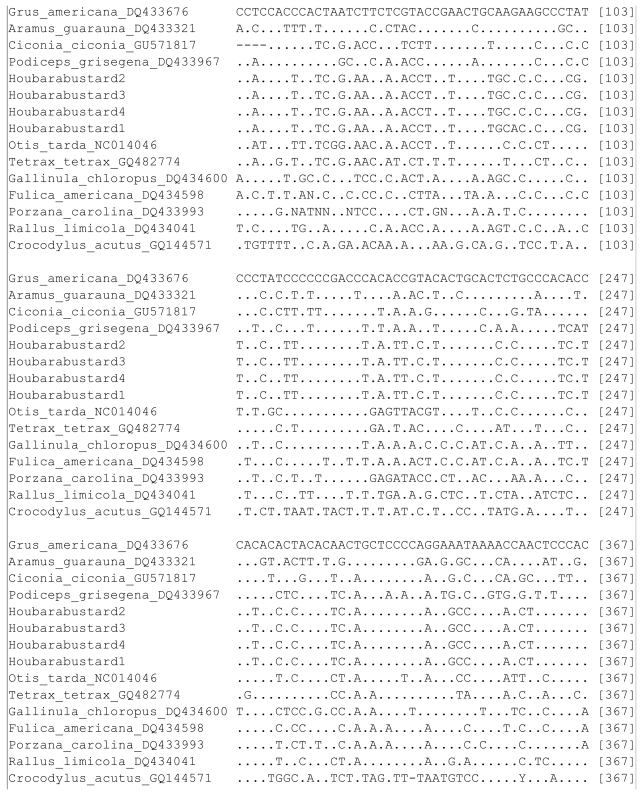

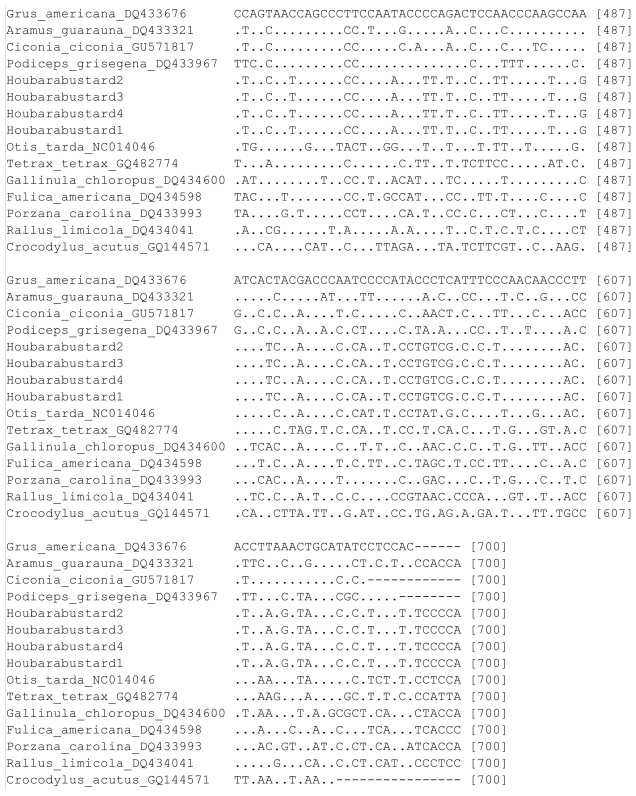
Haplogram of COI gene showing the variable sites among the 15 samples (12 species) representing different families of the orders Gruiformes, Ciconiiformes, Podicipediformes and Crocodylia (out group). The identical sites are represented by dots.

The nucleotide sequences of the mitochondrial COI gene segment (694 bp) from the four specimens of Houbara Bustard have been deposited in the GenBank with the accession numbers HQ168032 to HQ168035. Three of four of the specimens had an identical sequence of the COI gene, and one individual showed a single nucleotide difference (G > A transition at position 83). Within the family of bustards (Otididae), comparison between the three species (Houbara Bustard, *Otis tarda* and *Tetrax tetrax*), representing three different genera, showed 116 variable sites (Figure 2). For another family (Rallidae), the intra-family variable sites among the individuals of four different genera were found to be 146. Overall variable sites for the entire dataset of 15 individuals were 254 out of 700 total nucleotides (Figure 2). The COI genetic distances among the 15 individuals varied from 0.000 to 0.431 (Table 1).

**Figure 3:**
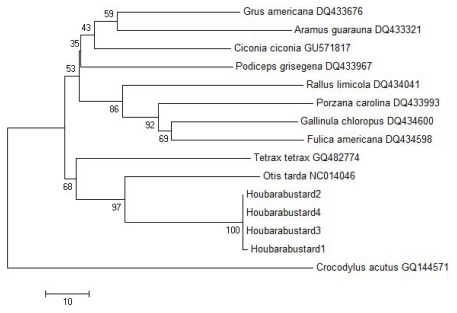
NJ tree showing the relationship among 15 different species including the out group, *Crocodylus acutus*.

**Figure 4:**
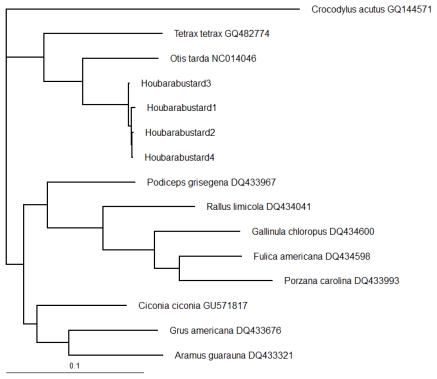
Bayesian tree showing the relationship among 15 different species, including the out group, *Crocodylus acutus*.

Phylogenetic analysis using neighbor-joining (NJ) (Figure 3) and Bayesian (BA) (Figure 4) methods discriminated among the species representing different orders, genera and families. Both trees classified the taxa into three clusters (excluding the out group); one of these clusters (family Otididae) was identical in NJ and BA trees. *Tetrax tetrax* and *Otis tarda* of the same family (Otididae) grouped with Houbara Bustard in the same cluster; *Otis tarda* with 90.43% similarity (Table 2) was placed closest to the Houbara Bustard in both the trees. All four specimens of Houbara Bustard formed a single clade. In NJ tree, all four species of the family Rallidae (*Rallus limicola*, *Gallinula chloropus*, *Fulica americana*, and *Porzana carolina*) grouped in a separate cluster with high bootstrap support for differentiating each genus (Figure 3). BA tree also placed all the species of Rallidae in one cluster (Figure 4). A separate cluster for the families Gruidae, Aramidae, Ciconiidae and Podicipedidae was observed in NJ tree (Figure 3). The only major difference between the NJ and BA trees was the placement of *Podiceps grisegena* (family Podicipedidae), which was grouped with the taxa of families Gruidae, Aramidae and Ciconiidae with low bootstrap support in NJ tree (Figure 3) or with the taxa of Rallidae in BA tree (Figure 4).

## 3. Discussion

Numerous efforts are being made to save the Houbara Bustard by captive breeding programs [22–26]. Wernery *et al*. [5] have demonstrated that Houbara gonadal primordial germ cells can migrate, differentiate and eventually give rise to functional sperm in chimeric chicken testis; this approach may provide a promising tool for propagation and conservation of endangered avian species that cannot breed in captivity. Although several studies have been undertaken to study biochemical, microbiological and immunological alterations in Houbara Bustard [23,27–30], limited data are available on molecular diversity of this bird. Idaghdour *et al*. [31] have sequenced the 854 bp segment of the mitochondrial control region from 73 birds to describe their population genetic structure with a particular sampling focus on the connectivity between *C. u. fuertaventurae* and *C. u. undulata*. Nucleotide and haplotypic diversity varied among the subspecies, the highest being in *C. u. undulata*, the lowest in *C. u. fuertaventurae* and intermediate in *C. u. macqueenii. C. u. fuertaventurae* and *C. u. undulata* are paraphyletic. Archaeological evidence indicates that Houbara Bustards have been present on the Canary Islands for 130,000–170,000 years. However, the genetic data point to a more recent separation of *C. u. fuertaventurae* and *C. u. undulata* at around 20,000–25,000 years [31].

The results of this study demonstrated the discriminatory power of COI barcodes for species identification as all the species appeared as separate clades of the phylogenetic tree. The sequences from the three samples of Houbara Bustard were found to be identical whereas only one within-species variable site was observed in the fourth sample of Houbara Bustard. In this study, all four samples were obtained from a captive breeding program, so a low level of intraspecific sequence variation was anticipated. However, it is worth mentioning that owing to its barcoding nature, within-species variation in COI gene is always low as compared with other phylogenetically important genes such as the mitochondrial control region (CR). In our recent study, the pair-wise sequence comparison of COI gene segment (692 bp) showed 53 (7.66%) variable sites across the three species of partridges, whereas within-species variable sites were found to be four (*Alectoris chukar*, four specimens), zero (*Alectoris philbyi*, two specimens) and three (*Alectoris melanocephala*, three specimens) [20]. In another study from our lab, the inter-specific genetic variation between the two species of bee-eaters was found to be 9.52%, whereas within-species (four specimens each) variable sites were found to be two (0.28%) and one (0.14%) for *Merops apiaster* and *Merops orientalis*, respectively using the same sized COI gene segment [21].

Previous research has suggested that Saudi Arabia contains both resident and migrant populations of Houbara Bustards [3]. However, the genetic diversity of Houbara Bustard in this region is still unknown. Phylogenetic analysis using nucleotide sequences of COI gene separated the 14 samples from five families into three different clusters using NJ (Figure 3) and BA (Figure 4) methods. The genera *Porzana*, *Gallinula* and *Fulica* of the family Rallidae appeared to be closer to each other but distant from the genus *Rallus* of the same family. The families Gruidae and Aramidae formed a common cluster with Ciconiidae and clearly separated from the family Rallidae (Figures 3 and 4). We used NJ and BA methods for creating phylogenetic trees that have been reported to be a better alternative to maximum parsimony method for phylogenetic inference using mitochondrial sequences [32]. Maximum likelihood and NJ methods have been shown to be nearly equally efficient and generally more efficient than the maximum parsimony method [33].

The mitochondrial protein-coding genes are regarded as powerful markers for genetic diversity analysis at lower categorical levels, including families, genera and species [34,35]. However, COI barcodes are able to identify taxonomic entities below the species level that may constitute separate conservation units [36]. Hebert *et al*. [15] have determined the COI barcodes for a large number of species of North American birds and found that all the species had a different COI barcode. Kerr *et al*. [16] have used COI barcodes for determination of intraspecific sequence divergences in eastern Palearctic birds. Yoo *et al*. [17] have applied COI barcodes for accurate discrimination of a large number of Korean birds. Fleischer *et al*. [37] have conducted DNA analysis of seven museum specimens of the endangered North American ivory-billed woodpecker and three specimens of the species from Cuba to determine their molecular diversity. Tavares *et al*. [38] have used a mitochondrial DNA COI barcode for investigating the population structure in water rails at the genetic level. A COI barcode amplified from a blood stain has been used for identification of the bird involved in the bird strike incident [39].

The above discussion clearly indicates that COI barcoding is a powerful tool for species identification and phylogenetic inference. In our dataset, the sequences of COI gene effectively discriminated different species, including the Houbara Bustard. However, caution must be exercised when using a single gene for inferring complex phylogenies. Although mitochondrial genes, including COI, provide phylogenetic information, conclusions from phylogenies based on a single locus have been questioned, because the resulting gene trees do not always recover species trees phylogenies [40]. The trees generated from the complete mtDNA genome showed greater resolution with high bootstrap support as compared with phylogeny inferred from individual genes [41]. A large number of nucleotide sites are needed to determine the whole-genome tree whereas a relatively small number of sites often results in a tree with closer topology [42]. It has been shown that blocks of contiguous sites are less likely to lead to the whole-genome tree than samples composed of sites drawn individually from throughout the genome [42]. Thus, for understanding complex phylogenetic relationships, the use of a complete mitochondrial genome should be preferred over a single gene locus. However, individual gene trees with conditional high bootstrap support may also provide useful phylogenetic information.

## 4. Experimental Section

Blood samples were collected from four Houbara Bustards that belonged to the captive breeding program of the NWRC at Taif, Saudi Arabia. We extracted DNA from blood samples using DNeasy Blood and Tissue Kit (Qiagen GmbH, Germany) in accordance with the manufacturer’s instructions. The DNA that we extracted was dissolved in 200 μL of elution buffer and stored at −20 °C.

COI sequences were amplified using the primer pair of BirdF1 and BirdR1 [12] and FideliTaq PCR master mix (GE Healthcare) in a reaction volume of 30 μL. The PCR conditions included a denaturation step (1 min at 94 °C) followed by six cycles of 1 min at 94 °C, 1.5 min at 45 °C, and 1.5 min at 72 °C, followed in turn by 35 cycles of 1 min at 94 °C, 1.5 min at 55 °C, and 1.5 min at 72 °C, and a final extension for 5 min at 72 °C. The PCR products were electrophoresed on a 1% agarose gel stained with ethidium bromide. The PCR products were purified using MicroSpin S300 columns (GE Healthcare) before being sequenced using BigDye Terminator Cycle Sequencing Kit (Applied Biosystems, USA) on 3130XL genetic analyzer (Applied Biosystems). For each sample, two sets of sequencing reactions were performed using the forward and reverse primers for high accuracy.

For evaluation of barcode sequences of this study with previously published sequence data of closely related species, we used the identification engine of the Barcode of Life Data (BOLD) website [43] to obtain the required information. We acquired 20 records from six species as follows: *Otis tarda* (one record), *Pygoscelis adeliae* (three records), *Ardeotis kori* (three records), *Ciconia ciconia* (four records), *Oceanites nereis* (five records), and *Podiceps grisegena* (four records) (Table 2). Two of these species belong to the same order (Gruiformes) and family (Otididae) as those of Houbara Bustard. Out of the total of six species shown in Table 2, the COI sequences of *Ardeotis kori* and *Oceanites nereis* are neither available in GenBank nor ready to download from BOLD, so we omitted them from our comparative study. The COI sequence of another species, *Pygoscelis adeliae*, reported in the GenBank, was quite short (456 bp) as compared to other species (≈700 bp), so this species was also removed from the final data set.

The family Otididae contains 10 genera, including *Otis*, *Ardeotis*, *Chlamydotis*, *Neotis*, *Eupodotis*, *Lophotis*, *Lissotis*, *Houbaropsis*, *Sypheotides and Tetrax*. Unfortunately, the COI barcode data of only two genera (*Otis* and *Tetrax*) are available in the GenBank, so we opted to include more families (instead of genera) of the order Gruiformes for a meaningful comparison. The Gruiformes is an order containing a considerable number of living and extinct families, with a widespread geographical diversity. Traditionally, a number of wading and terrestrial bird families that did not seem to belong to any other order were classified together as Gruiformes. These include 14 species of large cranes, about 145 species of smaller crakes and rails, as well as a variety of families comprising one to three species, such as the Heliornithidae, the limpkin, or the trumpeters. However, the COI barcodes of most of these families are yet to be established. The details of COI sequences retrieved from the GenBank for comparative evaluation with COI barcodes of Houbara Bustard are given in Table 3.

The sequences were aligned by ClustalW [44] and the alignment file was saved in MEGA format. The aligned sequence data were subjected to two different methods of phylogenetic reconstruction: (i) NJ and (ii) BA. The evolutionary distances were computed by the maximum composite likelihood method [45,46]. We used NJ and BA protocols because of their superiority to the maximum parsimony method for phylogenetic inference using mitochondrial sequences [32]. NJ analysis was performed using MEGA4 software and the bootstrap consensus trees inferred from 1000 replicates were taken to represent the evolutionary history of the taxa analyzed [47,48]. The Bayesian inference of phylogeny was conducted using MrBayes software [49] and the Bayesian trees were visualized with TreeView software [50].

## 5. Conclusions

The nucleotide sequence of partial segment of COI gene effectively discriminated different species, including the Houbara Bustard. This is the first study reporting the COI barcodes of Houbara Bustard. These data have multiple implications in forensic identification to curb illegal poaching, analyze molecular diversity, and as guidance for captive breeding programs.

## Figures and Tables

**Table 1:** Distance matrix of pair-wise sequence comparisons.

Sp.	GA	AG	CC	PG	HB1	HB2	HB3	HB4	OT	TT	GC	FA	PC	RL	CA
GA		68	68	76	81	81	81	82	83	78	87	91	82	90	124
AG	0.131		73	77	97	97	97	98	100	93	97	99	100	104	124
CC	0.133	0.145		70	74	74	74	75	89	84	90	86	82	87	129
PG	0.154	0.155	0.139		74	74	74	75	86	94	89	83	94	82	124
HB1	0.167	0.210	0.150	0.150		0	0	1	58	79	93	98	100	97	123
HB2	0.167	0.210	0.150	0.150	0.000		0	1	58	79	93	98	100	97	123
HB3	0.167	0.210	0.150	0.150	0.000	0.000		1	58	79	93	98	100	97	123
HB4	0.169	0.213	0.152	0.152	0.002	0.002	0.002		59	80	94	99	101	98	124
OT	0.170	0.218	0.189	0.180	0.106	0.106	0.106	0.108		78	95	96	98	98	131
TT	0.159	0.200	0.175	0.202	0.156	0.156	0.156	0.158	0.153		97	97	90	96	124
GC	0.187	0.216	0.193	0.192	0.203	0.203	0.203	0.205	0.206	0.216		59	68	76	138
FA	0.199	0.223	0.182	0.175	0.219	0.219	0.219	0.222	0.212	0.217	0.111		68	82	134
PC	0.172	0.223	0.171	0.205	0.224	0.224	0.224	0.227	0.216	0.197	0.130	0.130		91	147
RL	0.193	0.233	0.184	0.170	0.213	0.213	0.213	0.216	0.214	0.212	0.153	0.168	0.192		130
CA	0.312	0.315	0.340	0.316	0.310	0.310	0.310	0.313	0.336	0.309	0.383	0.368	0.431	0.342	

The upper panel shows the actual number of variable sites. The lower panel shows the number of base substitutions per site using the maximum composite likelihood model. Abbreviations are: Sp. (Species name); GA (*Grus Americana*); AG (*Aramus guarauna*); CC (*Ciconia ciconia*); PG (*Podiceps grisegena*); HB (Houbara Bustard); OT (*Otis tarda*); TT (*Tetrax tetrax*); GC (*Gallinula chloropus*); FA (*Fulica Americana*); PC (*Porzana Carolina*); RL (*Rallus limicola*); CA (*Crocodylus acutus*).

**Table 2:** List of closely-related species to Houbra Bustard, according to the Barcode of Life Data (BOLD) website search engine.

Class	Order	Family	Genus	Species	Similarity(%)
Aves	Gruiformes	Otididae	*Otis*	*tarda*	90.43
Aves	Sphenisciformes	Spheniscidae	*Pygoscelis*	*adeliae*	88.39
Aves	Sphenisciformes	Spheniscidae	*Pygoscelis*	*adeliae*	88.39
Aves	Sphenisciformes	Spheniscidae	*Pygoscelis*	*adeliae*	88.39
Aves	Gruiformes	Otididae	*Ardeotis*	*kori*	88.27
Aves	Gruiformes	Otididae	*Ardeotis*	*kori*	88.12
Aves	Gruiformes	Otididae	*Ardeotis*	*kori*	88.12
Aves	Ciconiiformes	Ciconiidae	*Ciconia*	*ciconia*	88.12
Aves	Ciconiiformes	Ciconiidae	*Ciconia*	*ciconia*	88.01
Aves	Ciconiiformes	Ciconiidae	*Ciconia*	*ciconia*	88.01
Aves	Procellariiformes	Hydrobatidae	*Oceanites*	*nereis*	88.01
Aves	Procellariiformes	Hydrobatidae	*Oceanites*	*nereis*	88.01
Aves	Procellariiformes	Hydrobatidae	*Oceanites*	*nereis*	88.01
Aves	Procellariiformes	Hydrobatidae	*Oceanites*	*nereis*	88.01
Aves	Procellariiformes	Hydrobatidae	*Oceanites*	*nereis*	88.01
Aves	Ciconiiformes	Ciconiidae	*Ciconia*	*ciconia*	87.83
Aves	Podicipediformes	Podicipedidae	*Podiceps*	*grisegena*	87.69
Aves	Podicipediformes	Podicipedidae	*Podiceps*	*grisegena*	87.69
Aves	Podicipediformes	Podicipedidae	*Podiceps*	*grisegena*	87.68
Aves	Podicipediformes	Podicipedidae	*Podiceps*	*grisegena*	87.66

**Table 3:** GenBank sequences used for comparative study.

GenBank Accession	Order	Family	Genus	Species	No. of Base Pairs
DQ434041	Gruiformes	Rallidae	*Rallus*	*limicola*	697
DQ434600	Gruiformes	Rallidae	*Gallinula*	*chloropus*	696
DQ434598	Gruiformes	Rallidae	*Fulica*	*americana*	697
DQ433993	Gruiformes	Rallidae	*Porzana*	*carolina*	695
DQ433676	Gruiformes	Gruidae	*Grus*	*americana*	681
DQ433321	Gruiformes	Aramidae	*Aramus*	*guarauna*	697
GQ482774	Gruiformes	Otididae	*Tetrax*	*tetrax*	694
NC014046	Gruiformes	Otididae	*Otis*	*tarda*	694
GU571817	Ciconiiformes	Ciconiidae	*Ciconia*	*ciconia*	648
DQ433967	Podicipediformes	Podicipedidae	*Podiceps*	*grisegena*	672
GQ144571 *	Crocodylia	Crocodylidae	*Crocodylus*	*acutus*	645

* Out group (American crocodile) from the Class Reptilia. The taxonomic classification of Houbara Bustard is: Order: Gruiformes; Family: Otididae; Genus: *Chlamydotis*; Species: *Chlamydotis undulate.*
